# Supplementary material for: Cyclic AMP signalling and glucose metabolism mediate pH taxis by African trypanosomes
Source: Nat Commun. 2022 Feb 1;13:603. doi: 10.1038/s41467-022-28293-w (PMC8807625; doi:10.1038/s41467-022-28293-w)
Supplement: Supplementary file 1 — Supplementary Information [file 41467_2022_28293_MOESM1_ESM.pdf]

Cyclic AMP signalling and glucose metabolism mediate pH taxis by African trypanosomes

Shaw et al.

Supplementary Figure 1

a

Plate	left	right
1	Alanine	Proline
2	Threonine	N-acetyl-D-glucosamine
3	Fumarate (di-Sodium salt)	NaOH
4	FeCl3	FeCl2
5	LiOH	NaOH
6	Calcium acetate	HCl
7	Ammonium hydrogen sulfate	Ammonium hydrogen carbonate
8	Calcium hydroxide	Magnesium hydroxid carbonate
9	Iron(II) sulfate	Ammonium iron(II) sulfate
10	Ammonium acetate	HCl
11	KOH	NaOH
12	Cadmium acetate	HCl
13	Tri-potassium citrate	HCl
14	Magnesium acetate	HCl
15	Copper sulfate	NaOH

b

Attractant	pH	Repellent	pH	no visible effect	pH
NaOH	14	HCl	1	Calcium hydroxide	12-13
LiOH	14	FeCl3	2	Ammonium acetate	7
KOH	14	FeCl2	2	Copper sulfate	3
Ammonium hydrogen carbonate	8-9	Iron(II) sulfate	2-3	N-acetyl-D-glucosamine	4-5
Magnesium hydroxid carbonate	9	Ammonium iron(II) sulphate	3		
Tri-potassium citrate	7-8	Ammonium hydrogen sulphate	1		
Calcium acetate	8	Sodium acetate	5-6		
Magnesium acetate	8	Cadmium acetate	7		
Proline	5	Alanine	5-6		
Fumarate (di-Sodium salt)	7	Threonine	5		

c

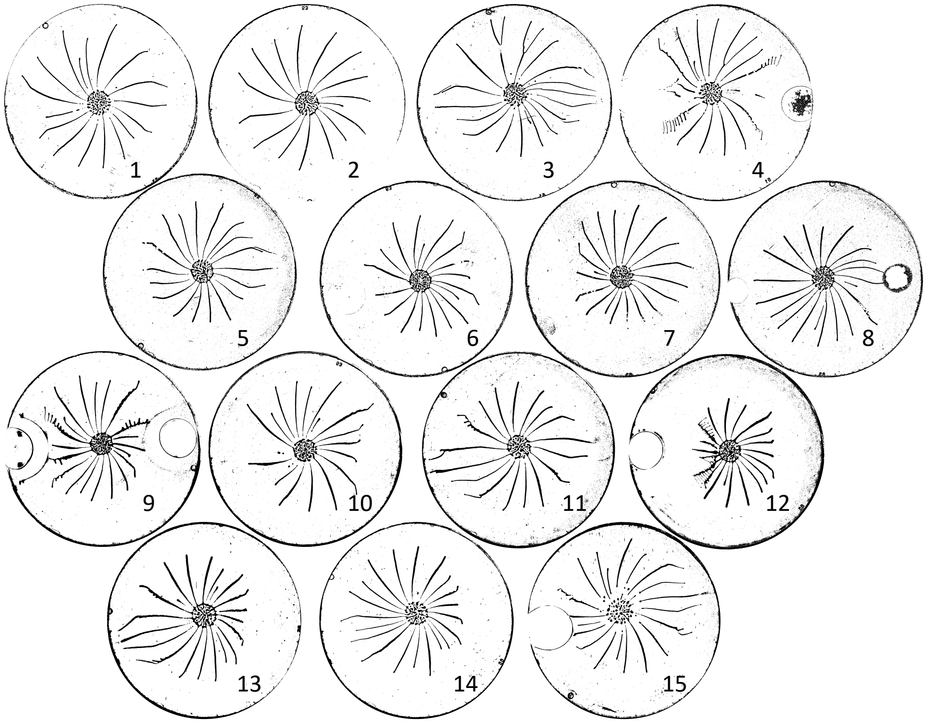

Supplementary Fig. 1 Different chemicals were tested for their impact on migrating cells on semi-solid surfaces. Procyclic forms of EATRO1125 were incubated on DTM plates. **a** All chemicals were dissolved or diluted in water to give 1 M solutions. Chemicals highlighted in red could not be dissolved completely, but were used anyway. **b** Effect on migrating cells and pH of 1M solutions. **c** SoMo assays. Chemicals were spotted on day 4 after inoculation and photographs were taken 16-20 h later. Photographs were processed to improve contrast and converted to black and white.

Supplementary Figure 2

a

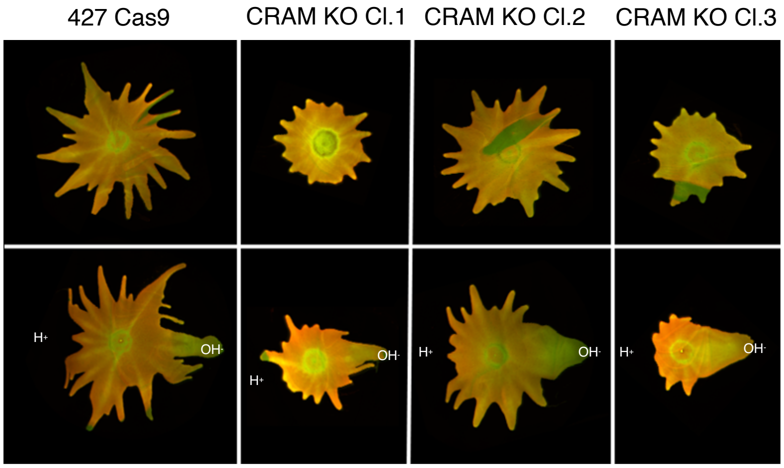

b

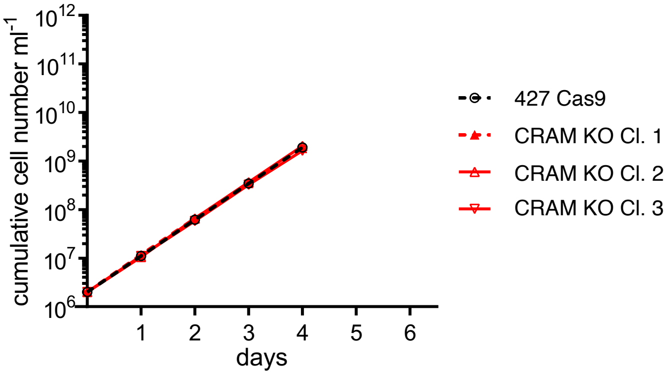

Supplementary Fig. 2 Independent CRAM knockout clones exposed to acid and alkali. **a** Upper panel: community lifts of CRAM knockout clones and their 427 Cas9 parental cell line. Lower panel: community lifts of knockouts and the parental line exposed to acid (H^+) and alkali (OH^-). The images show merged EP and GPEET signals. **b** Growth comparison of three individual CRAM knockouts and parental 427 Cas9. Source data are provided as a Source Data file.

Supplementary Figure 3

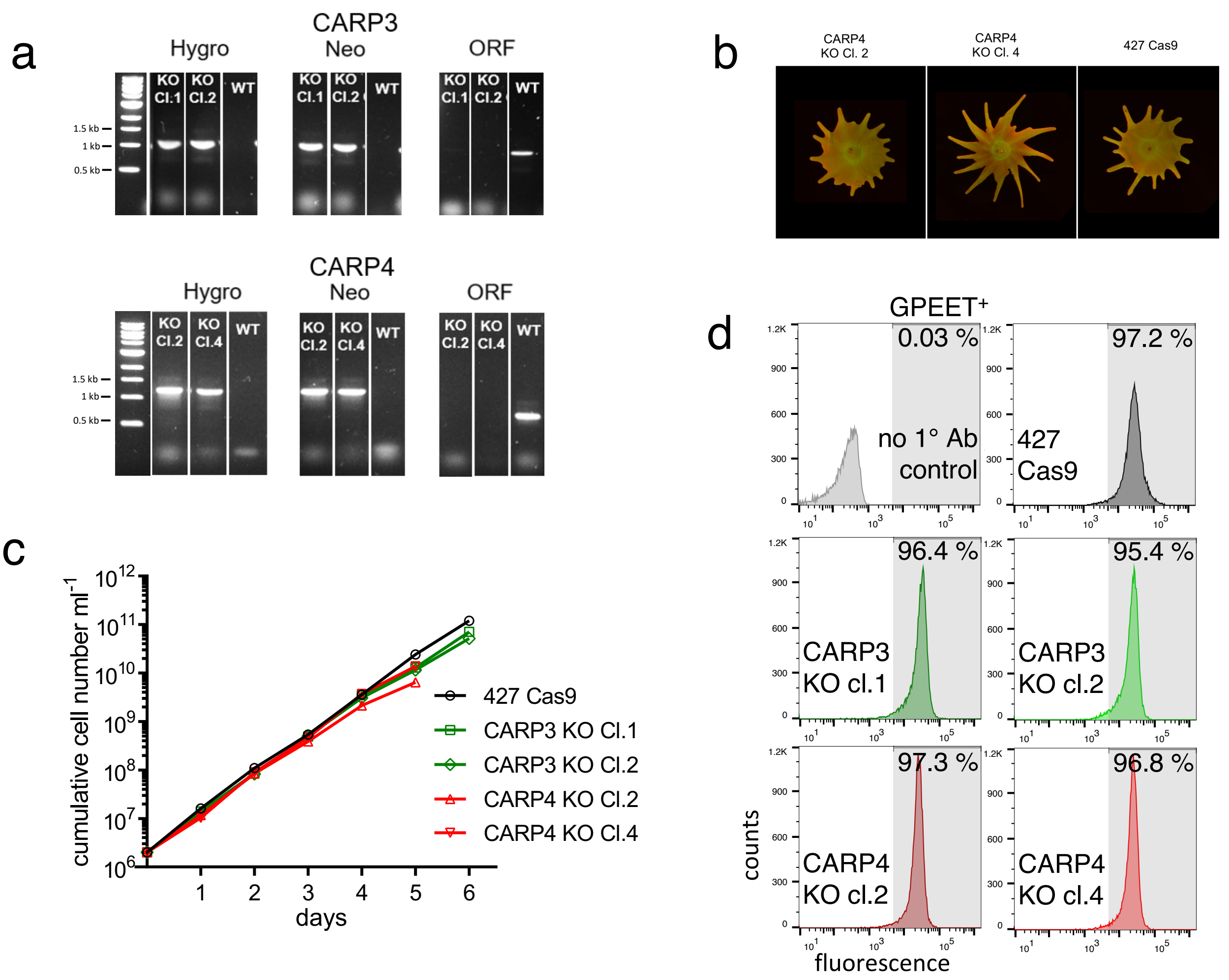

Supplementary Fig. 3 Characterisation of CARP3 and CARP4 knockouts. **a** Genotyping of CARP3 and CARP4 knockouts by PCR. Genomic DNA was isolated from individual clones and amplified with the primers listed in Supplementary Data file 3. **b** Community lifts of two individual CARP4 knockout clones. **c** Growth comparison of CARP3 and CARP4 knockouts and parental 427 Cas9. **c** Growth comparison of two individual CARP3 and CARP4 knockouts and parental 427 Cas9. **d** Percentage of GPEET-positive cells (shaded area) is indicated. Cells without prior incubation with anti-GPEET antiserum were used as a negative control (2° antibody only, grey). Source data are provided as a Source Data file.

Supplementary Figure 4

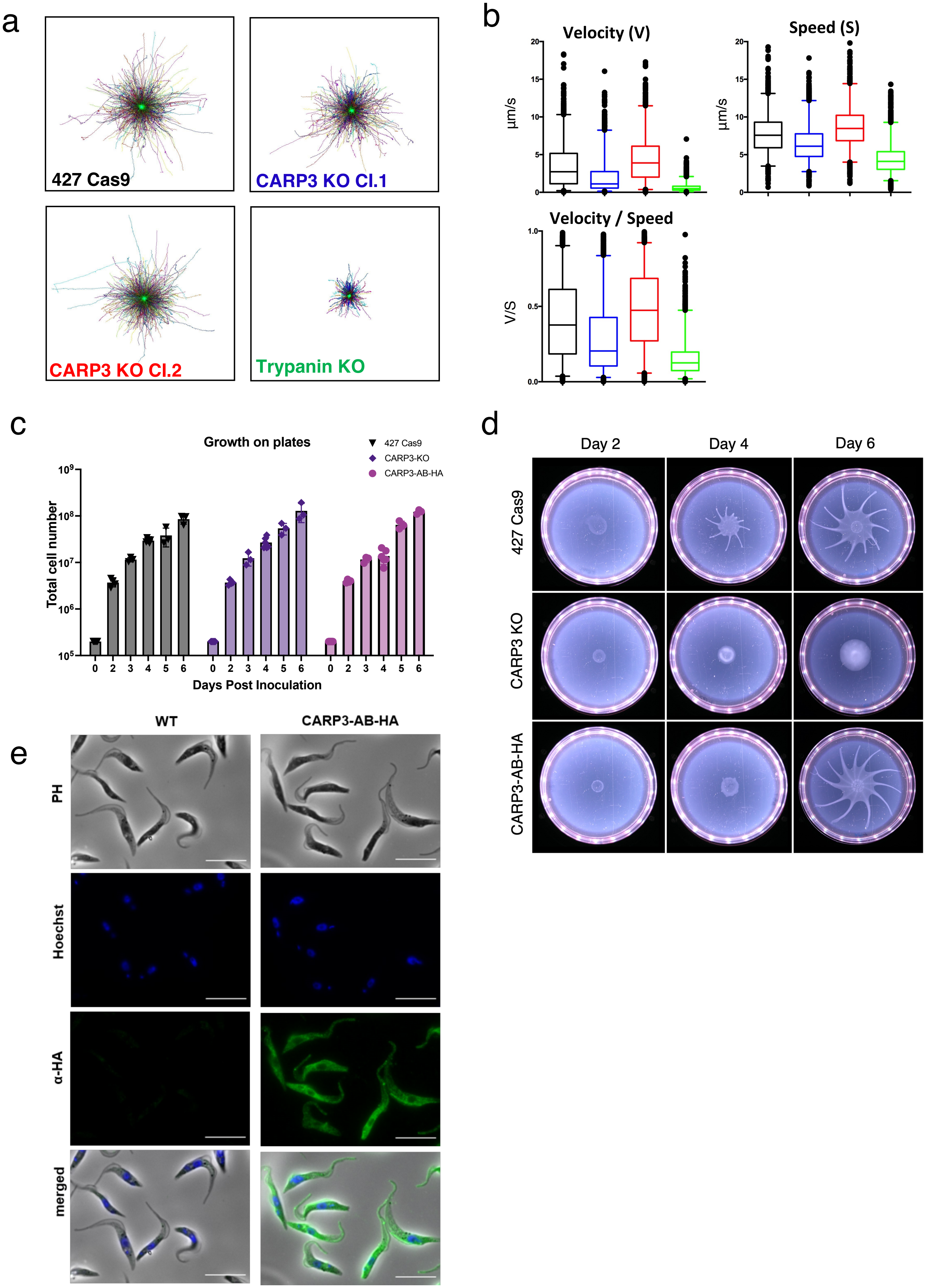

Supplementary Fig. 4 Motility and localization studies of CARP3 knockout. **a** Motility assay of CARP3 knockouts. Centred motility tracks of individual cells are shown at identical scale. > 8000 tracks for each cell line (427 Cas9, CARP3 KO Cl.1, CARP3 KO Cl.2 and Trypanin KO, respectively) were pooled from nine movies acquired from three technical replicates. The trypanin knockout was used as a motility-deficient control[1]. **b** Velocity and speed of each cell line. Tracks for individual cell lines 427 Cas9 (n = 2283), CARP3 KO Cl.1 (n = 2501), CARP3 KO Cl.2 (n = 2071) and Trypanin KO (n = 2606) respectively, were pooled from nine movies acquired from three technical replicates. The colour code corresponds to the cell lines in a. Data points are displayed as box plot with whiskers representing 2.5 - 97.5 percentile, filled circles represent points outside that range. InterQuartileRange IQR (25 - 75 percentile), with Median (50 percentile). **c** and **d** Determination of cell numbers on plates. Starting with an inoculum of 2×10^5 cells, cell numbers were determined for 427 Cas9, the CARP3 knockout and the HA-tagged addback on days shown in the figure. Cells were harvested by flushing the surface of the plate with PBS and cell numbers were determined using a haemocytometer. Cells from all three lines were observed to giggle under the microscope. n = 3 plates for days 3, 5 and 6; n = 4 plates for day 2; n = 6 plates for days 0 and 4. The plates were biologically independent. Data are presented as mean values +/- SEM. **e** Localisation of CARP3-HA (green) by immunofluorescence. DNA was stained with Hoechst dye. Scale bar = 10 μm. Source data are provided as a Source Data file.

Supplementary Figure 5

a

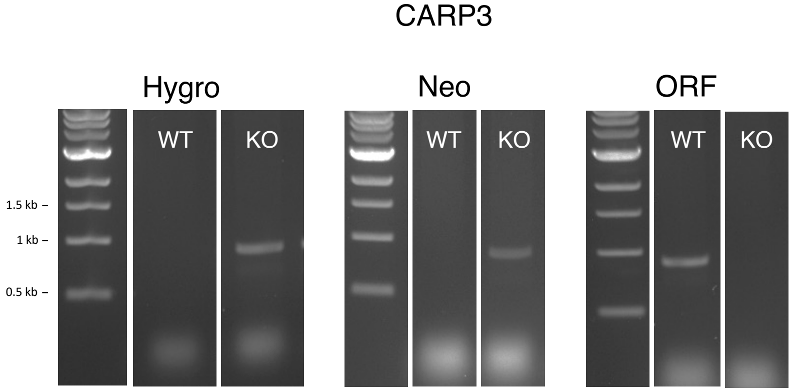

b

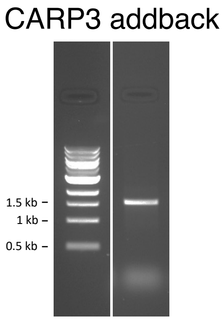

Supplementary Fig. 5 Genotyping of the CARP3 knockout (**a**) and addback (**b**) used for the fly experiments. Genomic DNA was isolated and PCRs were performed with the primers listed in Supplementary Data file 3. Source data are provided as a Source Data file.

Supplementary Figure 6

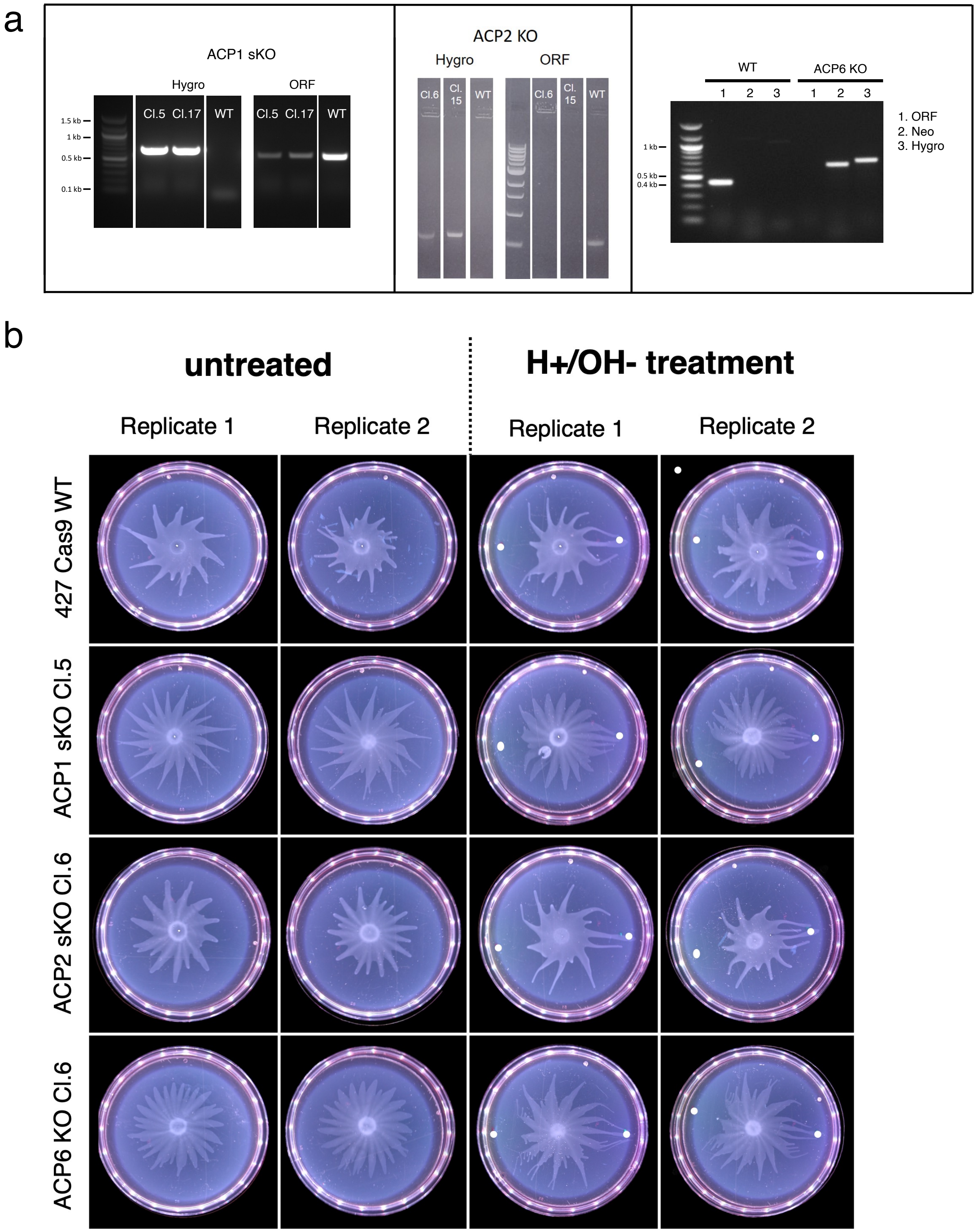

Supplementary Fig. 6 Characterisation of ACP1, ACP2 and ACP6 knockouts. **a** Genotyping of ACP knockouts by PCR. Genomic DNA was isolated and PCRs were performed with the primers listed in Supplementary Data file 3. **b** SoMo and pH taxis assays. Plates were inoculated with the cell lines indicated and 4 days later HCl and NaOH were applied to the plates (indicated by dots). Pictures were taken 16-20 h after spotting of chemicals. Source data are provided as a Source Data file.

Supplementary Figure 7

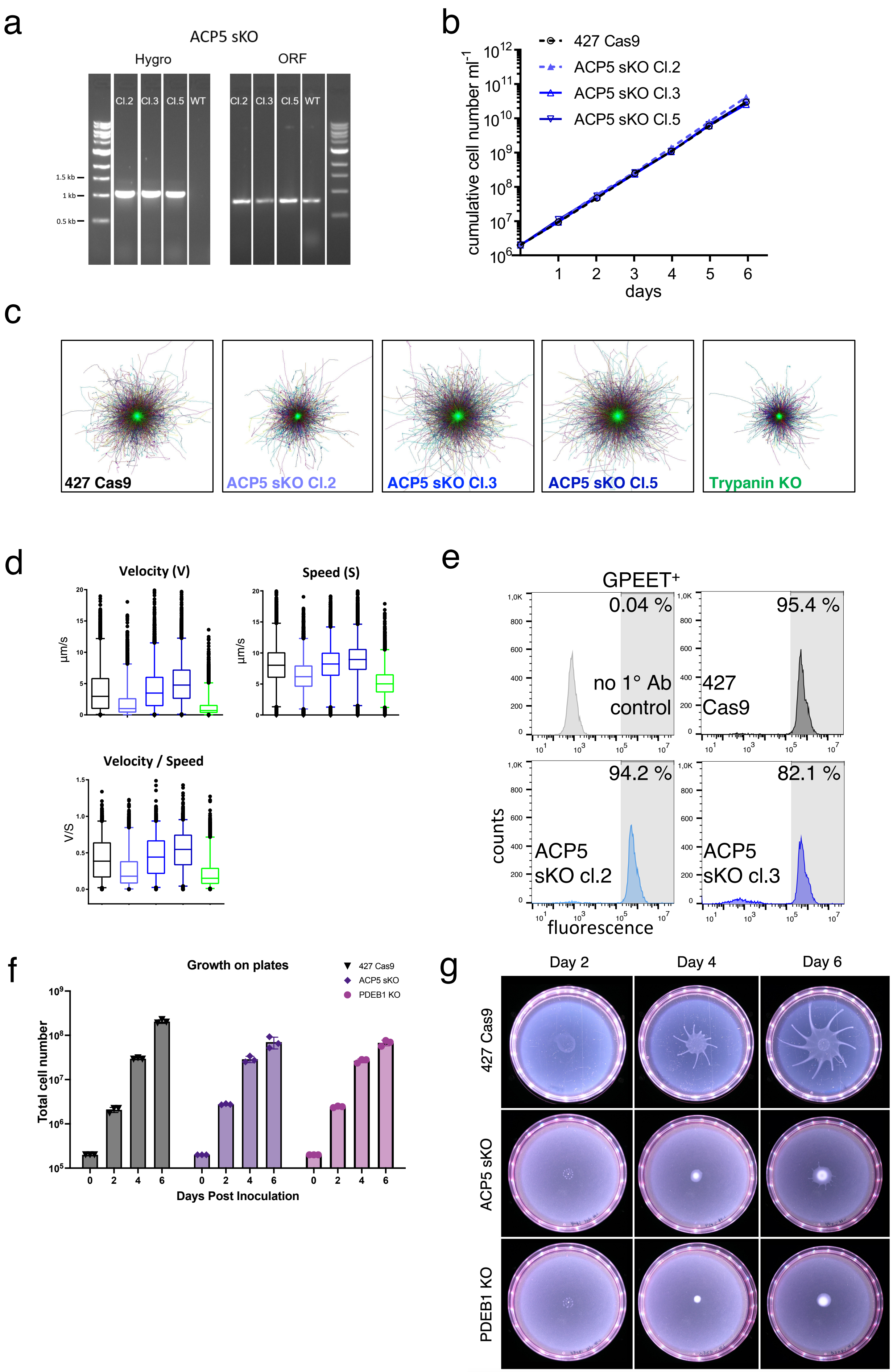

Supplementary Fig. 7 Characterisation of ACP1, ACP2 and ACP6 knockouts. **a** Genotyping of ACP5 single knockout by PCR. Genomic DNA was isolated and PCRs were performed with the primers listed in Supplementary Data file 3. **b** Growth of ACP5 single knockout clones compared to the parental 427 Cas9 cell line. **c** Motility assays of ACP5 single knockouts. Centred motility tracks of individual cells are shown at identical scale. >7000 tracks for each clone were pooled from nine movies acquired from three technical replicates. The parental line 427 Cas9 and Trypanin KO were used as controls. **d** Velocity and speed of individual cell lines. Tracks for individual cell lines 427 Cas9 (n = 8990), ACP5 sKO Cl.2 (n = 7030), ACP5 sKO Cl.3 (n = 9567), ACP5 sKO Cl.5 (n = 8171) and Trypanin KO (n = 8124) respectively, were pooled from nine movies acquired from three technical replicates. The colour code corresponds to the cell lines in c. Data points are displayed as box plot with whiskers representing 2.5 - 97.5 percentile, filled circles represent points outside that range. InterQuartileRange IQR (25 - 75 percentile), with Median (50 percentile). **e** Flow cytometric analysis of percentages of GPEET-positive cells. **f** and **g** Determination of cell numbers on plates. Starting with an inoculum of 2 x 10^5 cells, cell numbers were determined for 427 Cas9, the ACP single knockout and the PDEB1 knockout on days shown in the figure. Cells were harvested by flushing the surface of the plate with PBS and cell numbers were determined using a haemocytometer. n = 3 plates for each strain and each day. The plates were biologically independent. Data are presented as mean values +/- SEM. Source data are provided as a Source Data file.

Supplementary Table 1

a	Distance migrated after exposure (mm)			
	427 WT		PDEB1 KO	
	H+	OH-	H+	OH-
**Plate 1**	-1	15	1	2
**Plate 2**	-2	16	2	2
**Plate 3**	0	14	2	2
**Plate 4**	-2	14	0	2
**Plate 5**	0	13	1	2
**Average**	-1.0	14.4	1.2	2.0
**Stdev**	1.0	1.1	0.8	0.0

b	Distance migrated after exposure (mm)					
	427 Cas9		CARP3 KO		ACP5 sKO	
	H+	OH-	H+	OH-	H+	OH-
**Plate 1**	-1	20	0	8	0	10
**Plate 2**	-2	20	0	6	0	10
**Plate 3**	0	19	0	7	0	19
**Average**	-1.0	19.7	0.0	7.0	0.0	13.0
**Stdev**	1.0	0.6	0.0	1.0	0.0	5.2

Supplementary Table 1 Measurements of distances migrated by **a** 427 WT and PDEB1 knockout parasites and **b** 427 Cas9, CARP3 knockout and ACP5 single knockout parasites after exposure to acid and alkali. Parasites were inoculated as described for SoMo assays and incubated for four days. 1M HCl and NaOH (30 µl each) were added to the plates at a distance of 1.5 cm from the edge of the community for the PDEB1 knockout, CARP3 knockout and ACP5 single knockout and from the tips of the protrusions for 427 WT and 427 Cas9 (Fig. 3a, D = distance). The plates were sealed with parafilm and incubated at 27 °C for 16-20 hours. Positive numbers are migration towards and negative numbers are migration away from the spotted acid or alkali. Standard deviations were calculated from 5 or 3 samples respectively.

Supplementary Table 2

Experiment 1

**Midgut**	WT	KO	AB10
High	9	0	6
Intermediate	12	1	16
Low	10	5	4
Not infected	25	27	23
Total flies dissected	56	33	49
Infection rate (%)	55	18	53

**Proventriculus**	WT	KO	AB10
High	0	0	1
Intermediate	4	0	2
Low	1	0	3
Not infected	51	33	43
Total flies dissected	56	33	49
Infection rate (%)	9	0	12

Experiment 2

**Midgut**	WT	KO	AB10
High	5	0	12
Intermediate	9	0	3
Low	13	5	11
Not infected	69	106	61
Total flies dissected	96	111	87
Infection rate (%)	28	5	30

**Proventriculus**	WT	KO	AB10
High	0	0	4
Intermediate	1	0	5
Low	2	0	3
Not infected	93	111	75
Total flies dissected	96	111	87
Infection rate (%)	3	0	14

Supplementary Table 2 Fly infections. Midgut and proventriculus infection rates from two independent experiments with 427 Cas9, the CARP3 knockout and the untagged CARP3 addback.

Supplementary References

1. Shaw, S. *et al.* Flagellar cAMP signaling controls trypanosome progression through host tissues. *Nat. Commun.* **10**, (2019).
