# Supplementary material for: Cyclic AMP signalling and glucose metabolism mediate pH taxis by African trypanosomes
Source: Nat Commun. 2022 Feb 1;13:603. doi: 10.1038/s41467-022-28293-w (PMC8807625; doi:10.1038/s41467-022-28293-w)
Supplement: Supplementary file 3 — Description of Additional Supplementary Files [file 41467_2022_28293_MOESM3_ESM.pdf]

Description of Additional Supplementary Files

File Name: Supplementary Data 1
Description: RNAseq data. a Comparison of the roots and tips of projections. Transcripts from the tips either upregulated >1 Log2FC or downregulated 1 Log2FC in comparison to the root. FC: fold change. b Pairwise comparisons of untreated communities and communities exposed to acid or alkali. Transcripts from tips that were either upregulated >1 Log2FC or downregulated <1 Log2FC. FC: fold change. Analysis was done using DESeq2 bioconductor packag e to identify the differentially expressed genes. The package integrates Wald statistics to identify significantly regulated genes and adjustments were made for multiple comparisons with FDR/Benjamini-Hochberg. Raw read files are deposited at the European Nucleotide Archives (ENA) http://www.ebi.ac.uk/ena as study PRJEB41935.

File Name: Supplementary Data 2
Description: Proteins interacting with CARP3-HA, ACP3- Myc and ACP5-Myc identified by liquid chromatography – mass spectrometry (LC-MS).

File Name: Supplementary Data 3
Description: List of primers used for knockouts and genotyping.

File Name: Supplementary Movie 1
Description: Early procyclic form parasites exhibit social motility.

File Name: Supplementary Movie 2
Description: Response of early procyclic forms to hydrochloric acid (HCl). Black arrows indicate where solutions were spotted. The video is shown as a rewind loop.

File Name: Supplementary Movie 3
Description: Response of early procyclic forms to sodium hydroxide (NaOH). Black arrows indicate where solutions were spotted. The video is shown as a rewind loop.

File Name: Supplementary Movie 4
Description: Response of late procyclic forms to sodium hydroxide (NaOH). Black arrows indicate where solutions were spotted. The video is shown as a rewind loop.
