# Supplementary material for: Cyclic AMP signalling and glucose metabolism mediate pH taxis by African trypanosomes
Source: Nat Commun. 2022 Feb 1;13:603. doi: 10.1038/s41467-022-28293-w (PMC8807625; doi:10.1038/s41467-022-28293-w)
Supplement: Supplementary file 11 — Reporting Summary [file 41467_2022_28293_MOESM11_ESM.pdf]

nature portfolio

Corresponding author(s): Isabel Roditi

Last updated by author(s): Dec 23, 2021

Reporting Summary

Nature Portfolio wishes to improve the reproducibility of the work that we publish. This form provides structure for consistency and transparency in reporting. For further information on Nature Portfolio policies, see our Editorial Policies and the Editorial Policy Checklist.

Statistics

For all statistical analyses, confirm that the following items are present in the figure legend, table legend, main text, or Methods section.

n/a	Confirmed	
☐	☒	The exact sample size (*n*) for each experimental group/condition, given as a discrete number and unit of measurement
☐	☒	A statement on whether measurements were taken from distinct samples or whether the same sample was measured repeatedly
☐	☒	The statistical test(s) used AND whether they are one- or two-sided<br>*Only common tests should be described solely by name; describe more complex techniques in the Methods section.*
☒	☐	A description of all covariates tested
☒	☐	A description of any assumptions or corrections, such as tests of normality and adjustment for multiple comparisons
☐	☒	A full description of the statistical parameters including central tendency (e.g. means) or other basic estimates (e.g. regression coefficient) AND variation (e.g. standard deviation) or associated estimates of uncertainty (e.g. confidence intervals)
☐	☒	For null hypothesis testing, the test statistic (e.g. *F*, *t*, *r*) with confidence intervals, effect sizes, degrees of freedom and *P* value noted<br>*Give P values as exact values whenever suitable.*
☒	☐	For Bayesian analysis, information on the choice of priors and Markov chain Monte Carlo settings
☒	☐	For hierarchical and complex designs, identification of the appropriate level for tests and full reporting of outcomes
☒	☐	Estimates of effect sizes (e.g. Cohen's *d*, Pearson's *r*), indicating how they were calculated

Our web collection on statistics for biologists contains articles on many of the points above.

Software and code

Policy information about availability of computer code

Data collection	-Sequencing of cDNA libraries was performed at Fasteris, Geneva, using Illumina HiSeq sequencing systems with 150 bp read lengths and sequencing depths of approximately 20 million reads per sample.<br>-For motility assays, data was collected using Leica DM 5500B microscope<br>-Flow cytometry analysis was done using NovoCyte Flow Cytometer, model number: Novocyte 2100YB, ACEA Biosciences, Inc. (NovoExpress v1.2.5, ACEA Biosciences)
Data analysis	-Sequencing data was analyzed by using Galaxy (Version 2.11.40.6+galaxy1), an open source, web-based platform for data intensive biomedical research.<br>-The motiltiy assay dataset was analysed with a tracking macro provided by Richard Wheeler, University of Oxford. Velocity, speed and individual cell tracks were plotted using Fiji version 1.0 59 and Prism Version 6<br>-Flow cytometry data was analysed with FlowJo v7 and v10

For manuscripts utilizing custom algorithms or software that are central to the research but not yet described in published literature, software must be made available to editors and reviewers. We strongly encourage code deposition in a community repository (e.g. GitHub). See the Nature Portfolio guidelines for submitting code & software for further information.

Data

Policy information about availability of data

All manuscripts must include a data availability statement. This statement should provide the following information, where applicable:

- Accession codes, unique identifiers, or web links for publicly available datasets
- A description of any restrictions on data availability
- For clinical datasets or third party data, please ensure that the statement adheres to our policy

RNA sequencing raw data that support the findings of this study have been deposited in the European Nucleotide Archive (ENA) with the accession codes PRJEB41935 (Secondary Accession: ERP125782).

Field-specific reporting

Please select the one below that is the best fit for your research. If you are not sure, read the appropriate sections before making your selection.

[x] Life sciences [ ] Behavioural & social sciences [ ] Ecological, evolutionary & environmental sciences

For a reference copy of the document with all sections, see nature.com/documents/nr-reporting-summary-flat.pdf

Life sciences study design

All studies must disclose on these points even when the disclosure is negative.

Sample size	Numbers of flies used is based on the authors' knowledge of numbers sufficient to reveal a fly infection defect in known and published fly infection mutants (Ruepp et al., 1997 Journal of Cell Biology; Vassella et al., 2009 PLOSone; Shaw & DeMarco, 2019 Nat. Communications). Sample sizes only mattered for the fly experiments and no other experiments in this manuscript.
Data exclusions	No data exclusion
Replication	Multiple biological replicates were used for each experiment, as indicated in methods, supplemental methods and figure legends
Randomization	For fly infections, flies were allocated randomly into groups that were infected with wild type or mutant parasites.
Blinding	Blinding was not done to reduce any chances of mixing up cages of flies. People that dissected the flies had no expectations or preconceptions about the outcome. Data were only collated at the end of the experiment.

Reporting for specific materials, systems and methods

We require information from authors about some types of materials, experimental systems and methods used in many studies. Here, indicate whether each material, system or method listed is relevant to your study. If you are not sure if a list item applies to your research, read the appropriate section before selecting a response.

Materials & experimental systems

n/a	Involved in the study	
[ ]	[x]	Antibodies
[ ]	[x]	Eukaryotic cell lines
[x]	[ ]	Palaeontology and archaeology
[ ]	[x]	Animals and other organisms
[x]	[ ]	Human research participants
[x]	[ ]	Clinical data
[x]	[ ]	Dual use research of concern

Methods

n/a	Involved in the study	
[x]	[ ]	ChIP-seq
[ ]	[x]	Flow cytometry
[x]	[ ]	MRI-based neuroimaging

Antibodies

Antibodies used	- Mouse anti-EP: Anti-Trypanosoma brucei procyclin, Purified, (Clone TBRP1/247) (mouse IgG1), Product code: CLP001AP, Supplier name: Cedarlane<br>-Rabbit anti GPEET: Polyclonal anti-GPEET antibodies (K1) were raised in rabbits using a synthetic peptide, (GPEET)3C, coupled to KLH (Affiniti Research Products Limited, Nottingham, UK<br>-Monoclonal antibody 9E10 (Cat No: MA1-980) and Dynabeads™ Pan Mouse IgG (Cat No: 11041), both from ThermoFisher Scientific (Invitrogen).<br>-Monoclonal α-HA rat 3F10 (Cat. No: 11867423001) from Sigma Aldrich.<br>-Dilutions are given in the methods section for western blots, community lifts, flow cytometry and IFA. For co-immunoprecipitation the amount of antibody is relevant.
Validation	-Mouse anti-EP and Rabbit anti GPEET: Ruepp et al., 1997<br>-Monoclonal antibody 9E10: Manufacturer's statement: "This Antibody was verified by Relative expression to ensure that the antibody binds to the antigen stated."<br>-Dynabeads™ Pan Mouse IgG: Florini et al., 2019<br>-Monoclonal α-HA rat 3F10: Florini et al., 2019

Eukaryotic cell lines

Policy information about cell lines

Cell line source(s)	-Trypanosoma brucei Lister 427 (Bern stock) procyclic forms<br>-Procyclic forms of EATRO1125 were derived from bloodstream forms of T. b. brucei AnTat1.1<br>-Trypanosoma brucei 427 Cas9 (Shaw et al., 2019, Nat. Communications; Shaw et al., 2020, BMC Notes)
Authentication	Cells can be distinguished from other strains by morphology, expression profile of EP and GPEET and population doubling time. Individual strains can be distinguished with strain-specific PCR analysis.
Mycoplasma contamination	Cells were not tested for mycoplasma contamination
Commonly misidentified lines<br>(See ICLAC register)	No commonly misidentified lines used

Animals and other organisms

Policy information about studies involving animals; ARRIVE guidelines recommended for reporting animal research

Laboratory animals	Tsetse flies: Glossina morsitans morsitans
Wild animals	No wild animals were used.
Field-collected samples	No field-collected samples were used.
Ethics oversight	Ethics oversight was not required for fly experiments.

Note that full information on the approval of the study protocol must also be provided in the manuscript.

Flow Cytometry

Plots

Confirm that:

- [x] The axis labels state the marker and fluorochrome used (e.g. CD4-FITC).
- [x] The axis scales are clearly visible. Include numbers along axes only for bottom left plot of group (a 'group' is an analysis of identical markers).
- [x] All plots are contour plots with outliers or pseudocolor plots.
- [x] A numerical value for number of cells or percentage (with statistics) is provided.

Methodology

Sample preparation	GPEET measurement: Living Cells were spun down for 10 minutes, resuspended in 200 ul cold medium containing the primary antibody (polyclonal anti-GPEET antibody, K1, 1:1000) and incubated at 4°C for 30 minutes. After the incubation with the primary antibody, cells were washed twice with cold medium, then resuspended in 200 ul cold medium containing the secondary antibody (Alexa-green 488, 1:1000) and incubated at 4°C for 30 minutes. After the incubation with the secondary antibody, cells were washed twice with cold medium and resuspended in 1 ml 1x PBS and directly measured.
Instrument	NovoCyte Flow Cytometer, model number: Novocyte 2100YB, ACEA Biosciences, Inc.

Software	NovoExpress v1.2.5, ACEA Biosciences, Inc. and FlowJo v7 and v10.
Cell population abundance	No sorting was done
Gating strategy	No gating was performed since the samples only contained trypanosomes.

☒ Tick this box to confirm that a figure exemplifying the gating strategy is provided in the Supplementary Information.
